# Influence of Unit Cell Size and Fiber Packing on the Transverse Tensile Response of Fiber Reinforced Composites

**DOI:** 10.3390/ma12162565

**Published:** 2019-08-12

**Authors:** Royan J. D’Mello, Anthony M. Waas

**Affiliations:** Department of Aerospace Engineering, University of Michigan, Ann Arbor, MI 48109-2140, USA

**Keywords:** unit cell, fiber composites, crack band model, transverse tensile strength

## Abstract

Representative volume elements (RVEs) are commonly used to compute the effective elastic properties of solid media having repeating microstructure, such as fiber reinforced composites. However, for softening materials, an RVE could be problematic due to localization of deformation. Here, we address the effects of unit cell size and fiber packing on the transverse tensile response of fiber reinforced composites in the context of integrated computational materials engineering (ICME). Finite element computations for unit cells at the microscale are performed for different sizes of unit cells with random fiber packing that preserve a fixed fiber volume fraction—these unit cells are loaded in the transverse direction under tension. Salient features of the response are analyzed to understand the effects of fiber packing and unit cell size on the details of crack path, overall strength and also the shape of the stress-strain response before failure. Provision for damage accumulation/cracking in the matrix is made possible via the Bazant-Oh crack band model. The results suggest that the choice of unit cell size is more sensitive to strength and less sensitive to stiffness, when these properties are used as homogenized inputs to macro-scale models. Unit cells of smaller size exhibit higher strength and this strength converges to a plateau as the size of the unit cell increases. In this sense, since stiffness has also converged to a plateau with an increase in unit cell size, the converged unit cell size may be thought of as an RVE. Results in support of these insights are presented in this paper.

## 1. Introduction

Automation of the manufacturing process of composites may well be the next important cost efficiency gain in industrial applications (Beukers et al. [1]). Mass produced textile fiber performs that can be molded into near net shapes will lead to composite structures that have longer fatigue lives. There is an urgent need for robust and high fidelity computational tools that can replace costly testing associated with certification of composites for aerospace structural applications. Driven by the necessity of certification by analysis, integrated computational materials engineering (ICME) has emerged as a vibrant research activity in the past few years. Robust, mechanics based models that possess the correct physics at each stage of a production cycle will be needed to ensure maturity of ICME based computational frameworks. Summary of the major challenges related to ICME of composites can be found in Arnold et al. [2].

We focus on an important aspect of the ICME of fiber reinforced composites pertaining to composite unit cell at the micro-scale. Following earlier work (Heinrich et al. [3]), we address the issue of unit cell size that is needed for assessing the stiffness and strength of such unit cells when subjected to mechanical loads. The study includes virtual modeling that includes the development of damage and cracking as the unit cells are loaded. During processing of thermoset fiber reinforced polymer composites (FRPCs), the matrix undergoes shrinkage due to network formation and thermal strains due to thermal mismatch with surrounding fibers, which give rise to internal stresses. The state of the matrix at the end of curing can be altered by the presence of fibers and also by the form of the cure cycle. Song et al. [4] showed that the use of bulk matrix properties (i.e., measured “virgin” matrix properties) in numerical predictions of compression response of 2D triaxially braided composite representative volume element can lead to erroneous results. They reported that the computed compressive strength using the virgin matrix properties were higher than experimentally measured strength. Such a discrepancy in strength prediction using the so called “virgin” matrix properties arises because matrix that cures in the presence of the fibers is noticeably different from the virgin matrix. Such a matrix is called in situ matrix, which has the effective properties of the matrix that takes into account imperfections caused in the matrix during the curing process. The post-cure in situ matrix properties in terms of the matrix shear stress-shear strain response, can be extracted via an inverse analysis through uniaxial tensile loading of a ±45${}^{\circ }$ laminate, which is often convenient in engineering analysis of processed composites (Ng et al. [5]). The in situ shear stress-shear strain response can also be obtained from a torsion test. Thus, good knowledge of the state of matrix in the curing FRPC structure is important for ICME to be successful for FRPCs. Moreover, for a particular FRPC system, the optimal cure cycle must be identified such that the cured structure has the desired strength and stiffness. In the present investigation, we use micromechanics to study the influence of unit cells of certain size, which contain randomly packed fibers. The unit cells are chosen to be of different size (preserving the fiber volume fraction but containing different numbers of randomly packed fibers). The effects of curing of unit cells of different sizes is subject of ongoing work—that is, the presence of residual stresses in the composite due to processing is neglected in the present analysis.

**Figure 1:**
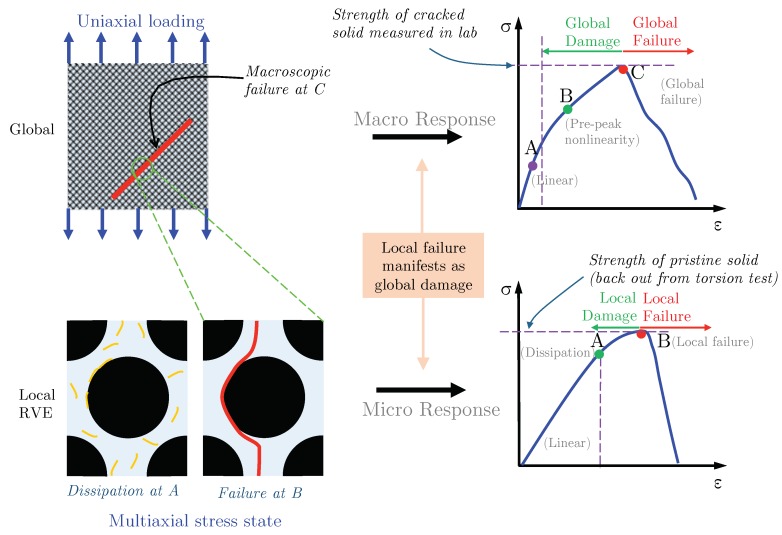
Qualitative behavior of the fiber matrix specimen (global) under uniaxial loading in contrast to behavior of fiber-matrix (local) constituents at the micro-scale.

Figure 1 qualitatively shows the behavior of a composite specimen at the global scale concurrently with the behavior of fiber-matrix constituents during initiation and propagation of damage up to subsequent failure. Although global damage is observed macroscopically, which manifests as matrix cracking resulting in a reduction of tangent stiffness, the matrix may have failed completely locally. Due to the inability to model the entire fiber-matrix microstructure at the component (global) level, the material properties at the global scale are usually the homogenized properties obtained from the constituents, using the properties of stiffness and strength from the unit cell. In a multiscale analysis setting using finite elements, a simple unit cell representation is often used to represent the effective behavior of the global structure at the finite element’s integration points. Therefore, it is important to first understand how the choice of the unit cell size affects its overall stiffness and strength representation of much larger volumes of fiber-matrix composite. Thus, the goal of this work is to show whether or not stiffness and strength of unit cells are controlled by its size.

In this study, the matrix tensile strength used is similar to what is representative of macroscopic coupon level measurements, while the Mode I fracture toughness $G_{IC}$ has been lowered by approximately two orders of magnitude compared to macroscopically measured values (for example, from a double cantilever beam test or a compact tension test with specimens in the tens of mm scale). The use of macroscopically observed values of $G_{IC}$ in the microscale simulations encountered in this study would lead to physically unrealistic response. Recently, Qiao and Salviato [6] proposed a two-scale cohesive traction-separation law. Based on measurements, they showed that the fracture strength of thermoset polymers at the microscale is noticeably higher than the macroscopically observed fracture strengths, whereas the fracture toughness, $G_{IC}$, associated with Mode I cracking is up to two orders of magnitude lower than what is observed in macroscopic specimens. Their observation has also been supported by coarse-grained molecular dynamics (MD) studies on the tensile behavior of thermoset polymers ( Yang and Qu [7]). This MD study showed that sharp reduction in cohesive strength occurs due to cavity initiation and coalescence phenomenon at the sub-micron scale associated with the crack-opening displacements. The subsequent macro-scale reduction in cohesive strength and toughness is due to re-alignment of the polymer chains in relatively less densely packed volumes. This suggests that the fracture toughness and strength measured at the macroscopic scale are not appropriate for use in microscale fracture simulations.

## 2. Modeling

**Figure 2:**
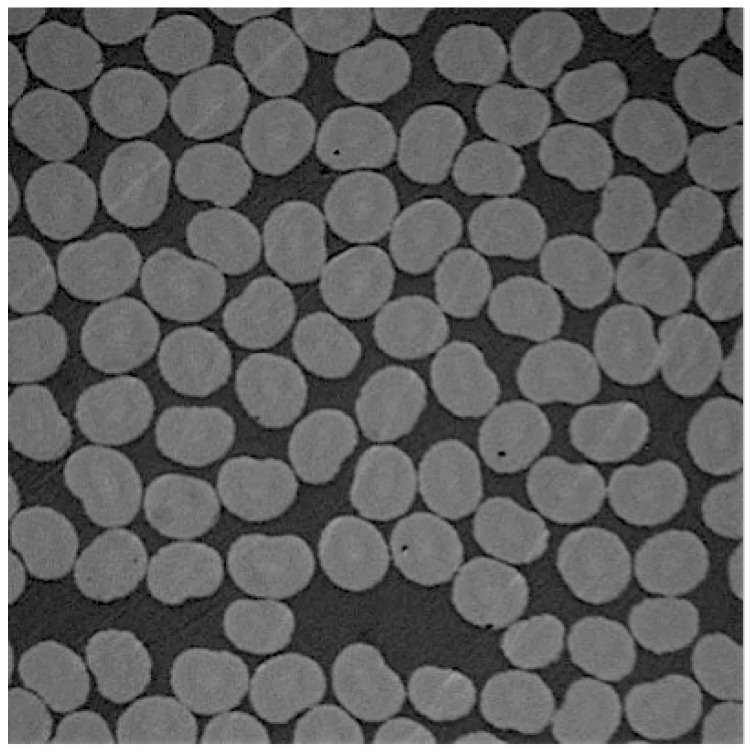
Random packing of fibers seen in a cross-section of a fiber tow with $V_{f}=0.68$.

In reality, the microstructure of fiber-reinforced composites exhibits randomness in packing (Figure 2), which in turn influences the mechanical properties (Bulsara et al. [8], Moulinec and Suquet [9]). In contrast, the baseline or the idealized case is the one where fibers are arranged in a hexagonally packed manner. One of the questions we can ask is that for a given fiber volume fraction, how much do the transverse fracture properties (strength and toughness) of the randomly packed configuration deviate from the perfect arrangement. Another aspect is determining the number of fibers (or unit cell size) for given (fixed) fiber volume fraction sufficient enough to represent the bulk transverse fracture properties. The problem of finding the smallest unit cell that will represent bulk fracture properties is affected more by the geometrical details of the constituents compared to corresponding problem for stiffness, where the bulk properties are governed by the average properties of the constituents.

**Figure 3:**
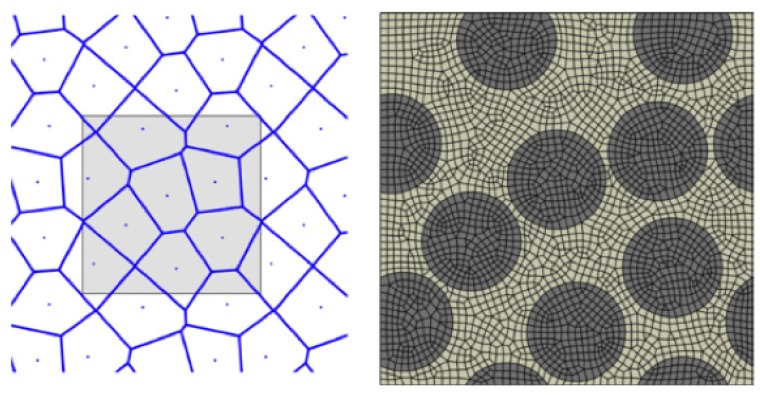
Voronoi cells and generated finite element model (Heinrich et al. [3]).

In an earlier work by Heinrich et al. [3] that addressed the influence of packing on the residual stress buildup during cure, the randomness in packing was characterized by Voronoi tessellation (Figure 3). The deviation from hexagonal packing was described in terms of the area of the Voronoi cells, the centers of which corresponded to the centers of fibers placed in the unit cell. The present investigation will follow a similar approach of characterizing fiber packing. For each unit cell size and for a given fiber volume fraction, several fiber packing renditions will be created.

**Figure 4:**
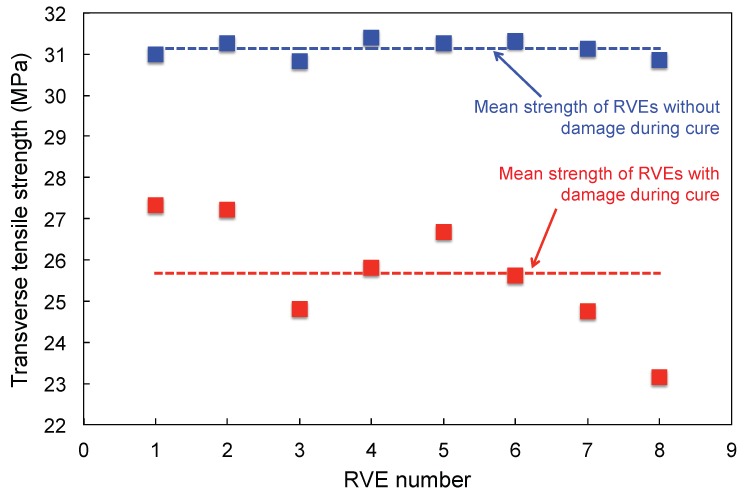
Variation in unit cell transverse tension strength (D’Mello et al. [10]).

Our prior work (D’Mello et al. [10]) on transverse loading of virtually cured randomly packed unit cells indicates that packing has a strong influence on transverse tensile strength (Figure 4). However, in this investigation, apart from influence of unit cell size, we also aim to find the influence of randomness in fiber packing and matrix fracture properties under transverse tension. Under tension, the crack band model described in the next section, will be used to model damage/failure propagation in the unit cells. The output from these simulations will provide transverse tension fracture properties—that is, unit cell strength and fracture toughness. Next step in the analysis is finding correlation between unit cell size (for a given fiber volume fraction) and the fracture properties and quantifying randomness associated with fracture properties. As shown in Figure 4, taken from D’Mello et al. [10], the effect of curing has a marked influenced on the unit cell strengths. This behavior is due to the presence of inherent self-equilibrating residual stresses in the unit cells on account of curing. When such unit cells are loaded in the transverse direction, the overall strain required for the matrix elements to enter failure is much lower. Moreover, the global stress recorded is also lower due to account of pre-existing tensile residual stress in the matrix.

**Figure 5:**
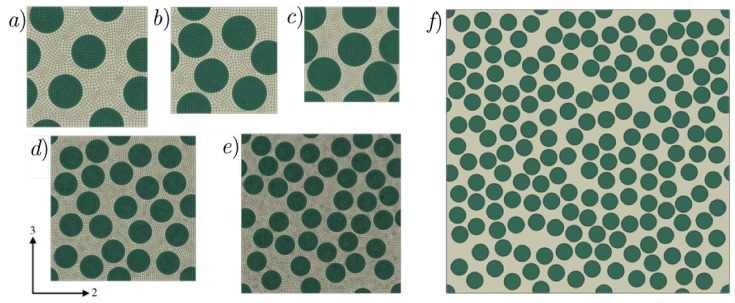
Types of randomly packed fiber unit cells: (**a**) six-fiber unit cell ($V_{f}$ = 0.4), (**b**) six-fiber unit cell ($V_{f}$ = 0.5), (**c**) six-fiber unit cell ($V_{f}$ = 0.6), (**d**) 24-fiber unit cell ($V_{f}$ = 0.5), (**e**) 40-fiber unit cell ($V_{f}$ = 0.5) and (**f**) 180-fiber unit cell ($V_{f}$ = 0.5). For each type of unit cell shown above, 10 different realizations of randomly packed fiber configurations are considered.

In the present work, random fiber unit cells with three different sizes are considered: unit cells having 6, 24, 40 and 180 fibers respectively. Each of these unit cells will be loaded in the transverse direction (two-direction) under tension. Commercially available finite element software ABAQUS/Explicit complemented by user subroutines are used for these numerical simulations. Each of the unit cells are modeled in 3D, using linear hexahedral C3D8 elements. The fiber diameter is taken to be eight microns and the dimension of the unit cell in the fiber-direction is kept fixed at 0.2 times the fiber diameter. The mesh density is taken such that the circumference of the fiber is modeled with approximately 38 C3D8 elements. For the unit cells with six fibers, three different fiber volume fractions ($V_{f}$) of 0.4, 0.5 and 0.6 are considered, to study the variation of fiber volume fraction on the stiffness and strength, whereas for the unit cells having 24, 40 and 180 fibers, a fiber volume fraction of 0.5 is considered in order to study the effect of size on the unit cell stiffness and strength. For each type of unit cell, 10 different realizations of randomly packed fiber configurations are considered to quantify the scatter in the mechanical response under transverse tension. Each type of unit cell used in the present work along with the coordinate frame indicated are shown in Figure 5. Carbon fibers are modeled as transversely isotropic solids with properties $E_{11}^{f}=$ 235,000 MPa, $E_{22}^{f}=E_{33}^{f}=$ 15,000 MPa, $G_{23}^{f}=7000$ MPa, $G_{12}^{f}=G_{13}^{f}$ = 27,000 MPa, $\nu_{23}^{f}=0.25$ and $\nu_{12}^{f}=\nu_{13}^{f}=0.2$. The matrix has Young’s modulus $E^{m}=4670$ MPa and Poisson’s ratio $\nu^{m}=0.35$. During tensile loading in the transverse direction (two-direction), the unit cells are subjected to flat boundary conditions, wherein the edges of the unit cell are allowed to expand or contract under loading but are constrained to remain flat throughout the loading process.

**Figure 6:**
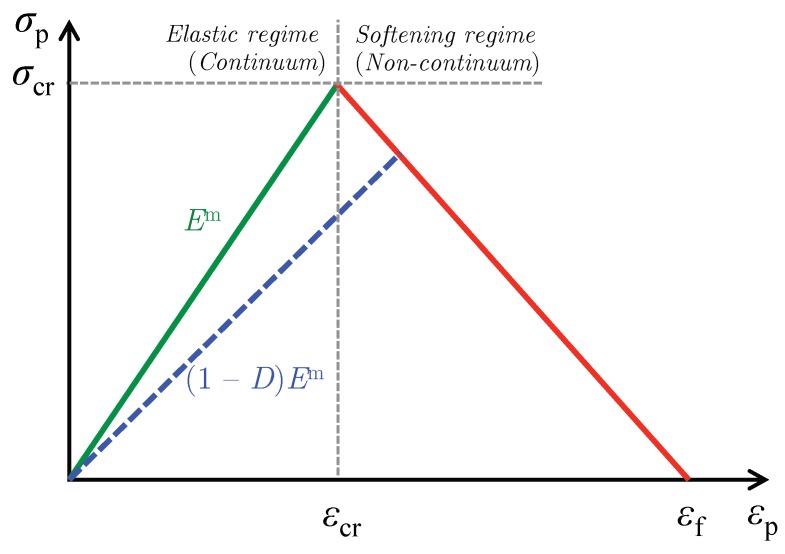
Crack band law in terms of maximum principal stress and maximum principal strain.

A crack band model based on the one proposed by Bazant and Oh [11] is used to model failure in the matrix. Methodology of crack band model for unit cells have been addressed in our earlier paper (D’Mello and Waas [12]) but will be repeated here for convenience. This model assumes that once the critical fracture stress $\sigma_{cr}$ is reached locally in the matrix, microcracks are formed and this effect is smeared over an element. The maximum principal stress criterion is used to determine the failure initiation in the matrix. However, other failure criteria can also be used for transverse compression, based on experimental observations and data that is relevant for modeling transverse compressive failure. For example, Pineda et al. [13] have used Mohr–Coulomb failure criterion for compressive-shear failure in fiber-reinforced composite unit cells using finite element analysis as well as by using high-fidelity generalized method of cells (Aboudi et al. [14]). In the present study for unit cells under transverse tension, when the maximum principal tensile stress in the matrix exceeds the critical value $\sigma_{cr}$, the traction–separation law controls the behavior of the damaging material as shown in Figure 6 and the stiffness of the matrix is reduced and consequently tracked using the secant modulus. When a traction–separation law controls the behavior of the damaging solid, the response is that of a non-continuum in the softening regime. Under mode I cracking, the energy dissipated during failure is the critical mode I energy release rate $G_{IC}$, given by

(1) $$G_{IC}=\int_{0}^{\delta_{f}}\sigma_{p}\left(\delta \right)d\delta =h\int_{0}^{\varepsilon_{f}}\sigma_{p}\left(\varepsilon_{p}\right)d\varepsilon ,$$

where, $\sigma_{p}$ and $\varepsilon_{p}$ is the maximum principal stress and principal strain, respectively, the maximum separation $\delta_{f}=h\varepsilon_{f}$ where $\varepsilon_{f}$ corresponds to the strain corresponding to complete failure of the material (accompanied by complete loss of stiffness). Here, *h* is the characteristic element length that preserves mesh objectivity (see Jirasek and Bazant [15]), by prescribing a normalized value of $G_{IC}$ for each element such that $g_{IC}=G_{IC}/h$. Consequently, the value of $g_{IC}$ equals the area under the $\sigma_{p}-\varepsilon_{p}$ law as shown in Figure 6. In the finite element procedure, the finite element sizes were kept more or less uniform in all the unit cells considered. The characteristic length *h* is computed for each element via Abaqus’s internal characteristic element length CELENT variable (Abaqus [16]). The value of $G_{IC}$ was chosen to be 0.002 N/mm in all the computations, whereas the tensile fracture strength $\sigma_{cr}$ was chosen to be 55 MPa. From the crack band model formulation, the damage parameter due to softening is denoted as *D* where 0≤D≤1. For a material with initial matrix stiffness $E^{m}$ now in the softening region of the traction-separation law, *D* is computed as:

(2) $$D=1-\frac{\sigma_{cr}}{E(\varepsilon_{f}-\varepsilon_{cr})}\left(\frac{\varepsilon_{f}}{\varepsilon_{p}}-1\right),$$

where $\varepsilon_{p}$ is the current maximum principal strain. Thus, the case D=0 corresponds to no damage in the finite element, the case 0<D<1 corresponds to damage but no two-piece failure in the element, while the case D=1 indicates failure with complete loss of stiffness. Thus, the quantity 1−D is a measure of stiffness reduction in the damaging matrix with as a fraction of the original stiffness $E^{m}$. Note that although we are dealing with a 3D solid, the stress–strain equation for the traction separation law is provided in the 1D form because the relevant stress and strains are assumed to be arising from an equivalent definition of stress and strain. In this case, the equivalent stress and strains are taken as maximum principal values as described in Bazant and Oh [11]. The damage parameter *D* is calculated based on this assumption and is used to degrade the 3D solid.

The notion of an representative volume elements (RVEs) is most appropriate for finding stiffness (elastic) properties of the bulk. However, once failure has set in (i.e., in the regime past the peak in the stress–strain response of the unit cell), the concept of an RVE in the traditional sense is lost. Failure in realistic bulk materials occurs in a localized manner. Thus, we can no longer assume that failure process is replicated in all periodic arrays of material in the bulk. The existence and determination of RVE has been addressed in detail by Gitman et al. [17], who demonstrated that an unit cell cannot be found for a material in the softening regime. Later, Nguyen et al. [18] have also addressed the issue of existence of the unit cells for softening quasi-brittle materials with random microstructure.

It is also instructive to point out and differentiate the quantities related to various sizes used in the finite element simulations relevant to the discussion of the crack band model. First is the typical length $L_{d}$ of the elements that must be maintained in order to achieve stress convergence, especially in zones where stress gradients are high, such as in the vicinity of fibers. In the present study, $L_{d}$ is proportional to circumferential length of the fibers, that is $L_{d}\propto 2\pi R_{f}$, where $R_{f}$ is the fiber diameter. This length is independent of the actual length scale of the structure but prescribed in proportion to fiber geometry, so that the computed stresses around fibers have converged. Next, independent of $L_{d}$, there are additional lengths $L_{I}$ and $L_{II}$ which must ensure that the tangent strain–softening modulus, for modes I and II cracking respectively, associated with the crack band model is not negative. These two lengths depend on the length scale of the element. Here, $L_{I}<\frac{2EG_{IC}}{\sigma_{cr}^{2}}$ for mode I cracking and $L_{II}<\frac{2GG_{IIC}}{\tau_{cr}^{2}}$ for mode II cracking, where *G* is the shear modulus and $\tau_{cr}$ is the critical shear strength for mode II cracking. For use in finite elements, recommended limits for $L_{I}$ and $L_{II}$ are half of what is prescribed by the expressions. Although stresses may have converged with the use of converged $L_{d}$, we must ensure that $L_{d}<L_{I}$ and $L_{d}<L_{II}$, so that the size of elements are suitable for use in the crack band model.

## 3. Results

### 3.1. Influence of Unit Cell Size on Transverse Stiffness and Strength

**Figure 7:**
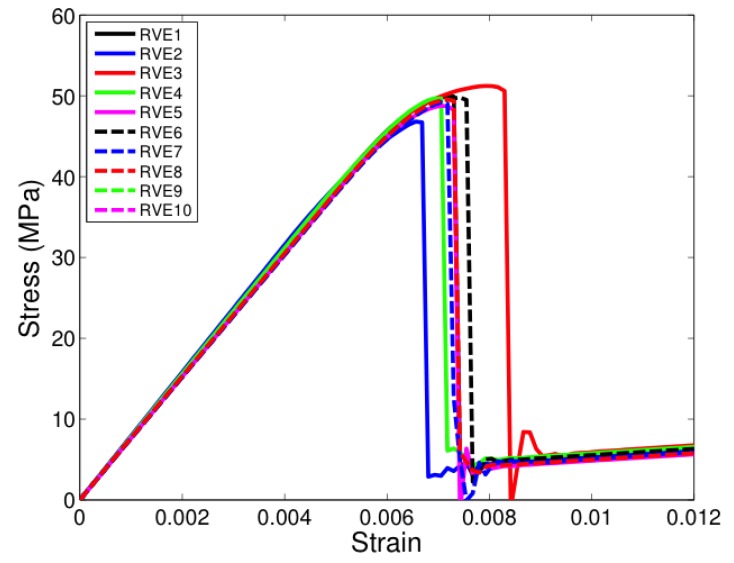
Transverse tensile response of six-fiber unit cells with $V_{f}$ = 0.4. (D’Mello and Waas [12]).

**Figure 8:**
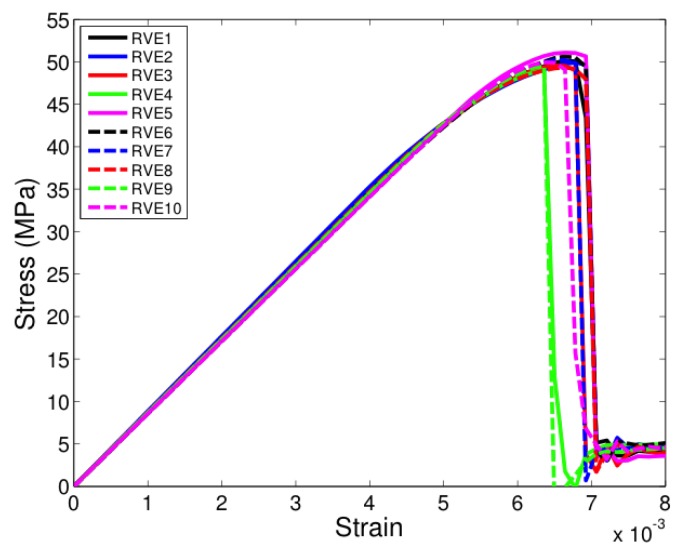
Transverse tensile response of six-fiber unit cells with $V_{f}$ = 0.5. (D’Mello and Waas [12]).

**Figure 9:**
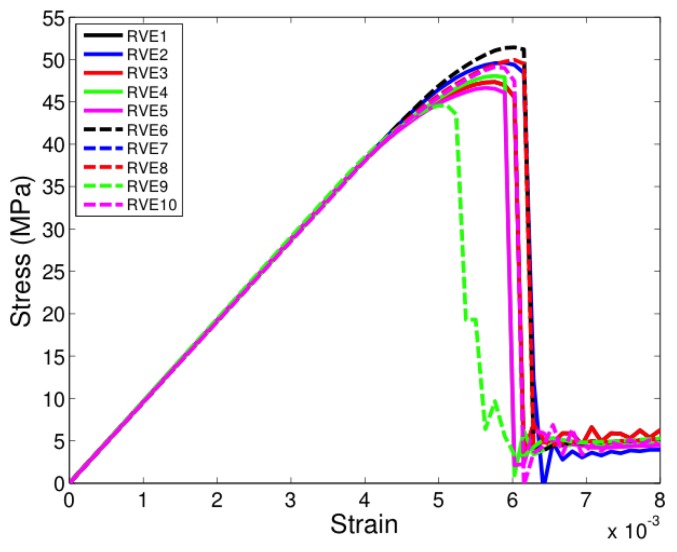
Transverse tensile response of six-fiber unit cells with $V_{f}$ = 0.6. (D’Mello and Waas [12]).

**Figure 10:**
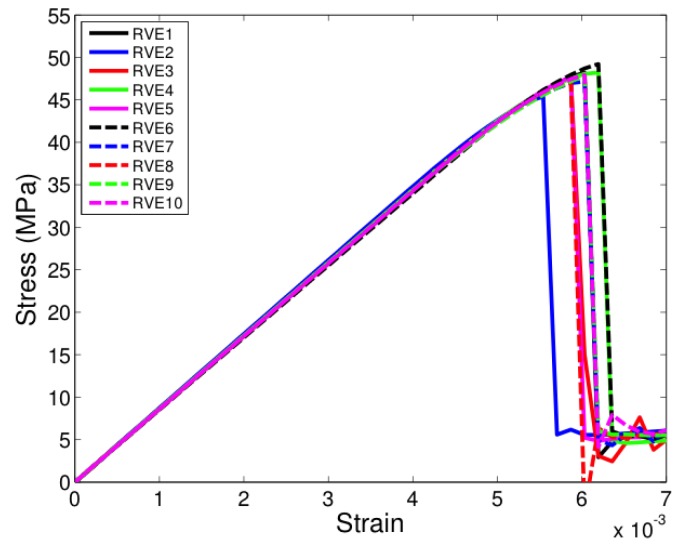
Transverse tensile response of 24-fiber unit cells with $V_{f}$ = 0.5. (D’Mello and Waas [12]).

**Figure 11:**
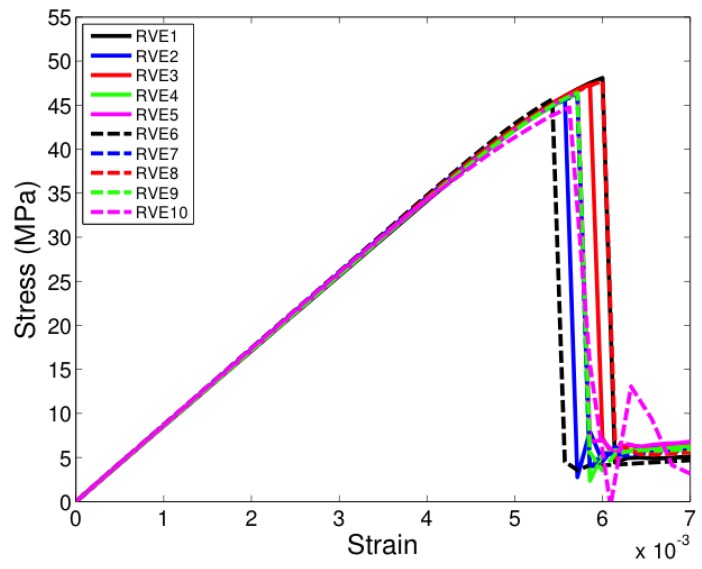
Transverse tensile response of 40-fiber unit cells with $V_{f}$ = 0.5. (D’Mello and Waas [12]).

All the unit cells were loaded under tension in the two-direction, that is, along a direction transverse to direction of fibers seen in Figure 5. Figure 7, Figure 8 and Figure 9 show the stress-strain response of six-fiber unit cells for fiber volume fractions 0.4, 0.5 and 0.6 respectively. Figure 10 and Figure 11 show the stress–strain response of 24-fiber and 40-fiber unit cells with fiber volume fraction 0.5. All these response plots exhibit similar behavior except that there is a scatter in the peak stress values owing to effect of fiber packing. Fiber packing affects the stress–strain response in that the development of tensile stresses in different regions is governed by the manner in which the fibers are distributed within the unit cell, hence the scatter. During the initial stages of loading, the unit cell exhibits a linear response followed by a peak and the response is governed only due to the elastic properties of the constituents. Before the peak is attained, there is some nonlinearity in the response caused when some of the matrix elements reach their critical tensile strength and enter the softening regime. When sufficient matrix elements enter the softening regime, a sharp two-piece crack develops. This crack is oriented approximately perpendicular to the loading direction and the formation of this crack controls the peak of the stress–strain response of each of the unit cells. In the elements that were in the softening path of the traction–separation law, the minimum stiffness was not allowed to fall below 2% of the original stiffness in the simulations to prevent excessive distortion in the elements. Therefore, the slight stress increase seen after the drop is a modeling artifact and not relevant to the analysis after the drop has occurred beyond the peak.

**Figure 12:**
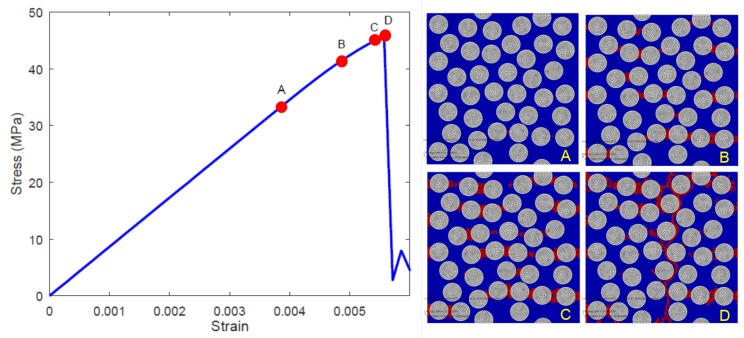
Matrix elements entering crackband in a realization of 40-fiber unit cell (#2) that cause nonlinearity starting at (*A*) through (*B*) and (*C*) but just before the peak at (*D*) (**left**); Complete failure in the matrix elements that is reflected as a sharp vertical crack in the unit cell (**right**) past the peak (*D*).

Figure 12 shows one of the realizations (unit cell #2 with $V_{f}=0.5$) of the 40-fiber case and the snapshots of the damage initiation (*A*) for transverse nominal strain 0.004. Upon further loading, damage accumulation (B,C) is seen to occur in the matrix regions, predominantly in the region between fibers. This damage accumulation has an effect of reducing the unit cell’s tangent stiffness. This softening response in few elements gives rise to the shorter nonlinear behavior in the stress-strain response just before the peak (*D*), followed by a sharp drop past the peak. Note that in the present model, there is no provision for fiber–matrix interface debonding, because it is assumed that fiber–matrix interface strength is higher than matrix tensile strength. Nonetheless, the two piece crack path is seen to traverse around the fibers and propagate through the thickness of the unit cell (i.e., along the three-direction). For other unit cells of similar size, the actual crack path was different, owing to the difference in fiber packing, however the general features of damage initiation, propagation and development of final crack are similar to what is seen in Figure 12.

**Figure 13:**
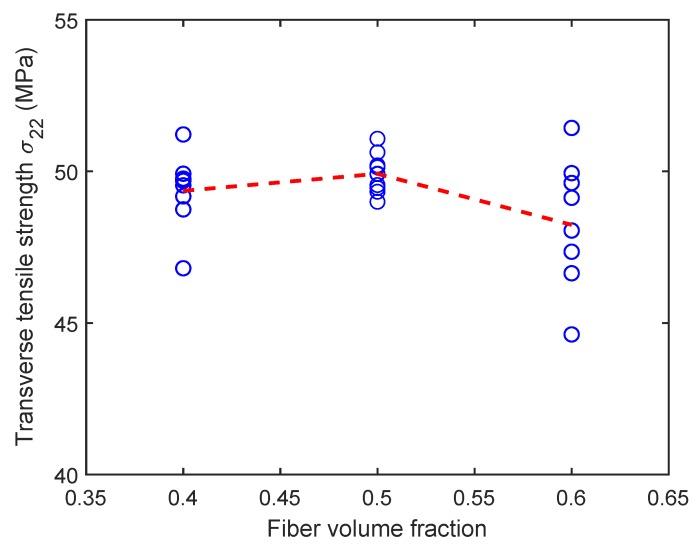
Effect of fiber volume fraction on the transverse tensile strength for six-fiber unit cells. (D’Mello and Waas [12]).

**Figure 14:**
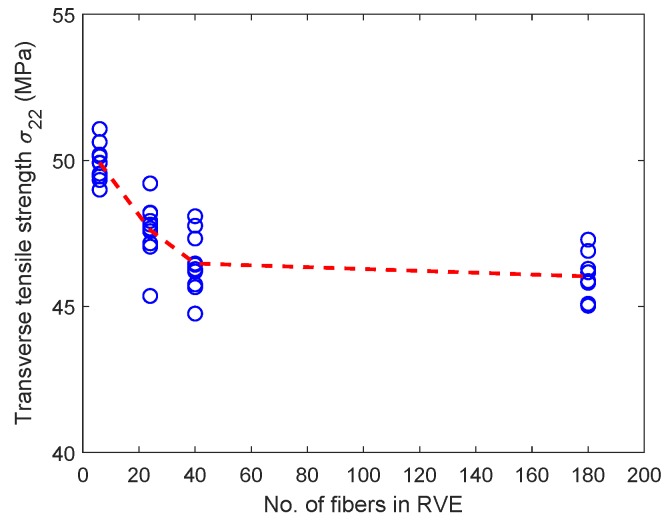
Variation of transverse tensile strength with unit cell size with $V_{f}$ = 0.5. (D’Mello and Waas [12]).

Figure 13 shows the variation of tensile strength for the six-fiber unit cell case but for varying fiber volume fractions. Although there is scatter in tensile strength in these unit cells, the mean unit cell strength appears to be insensitive to the fiber volume fraction. Figure 14 shows the variation of tensile strength of the unit cells of different sizes when the fiber volume fraction of the unit cell is held constant at 0.5. The mean transverse strength decreases with increase in unit cell size and then appears to stabilize, as can be seen in only a marginal reduction in the mean transverse strength between 40-fiber and 180-fiber renditions. Thus, increasingly larger unit cell sizes have to be considered to evaluate the size at which the transverse tensile strength value converges. The total volume of a unit cell of side length *L* scales as $L^{3}$, while the band volume within which dissipation occurs scales as $w_{cb}L^{2}$ where $w_{cb}$ is the width of the process zone. Thus, the ratio of dissipation volume to total volume is $\frac{w_{cb}}{L}$. Since $w_{cb}$ is small and a fixed material property for all cases, the scaling is inversely proportional to the path of the band, suggesting that larger heights of unit cells (and more stored energy) will require more than the available crack path length for complete dissipation. Therefore, larger unit cells will fail rapidly (unstably) and will release less energy than stored at fracture initiation.

**Figure 15:**
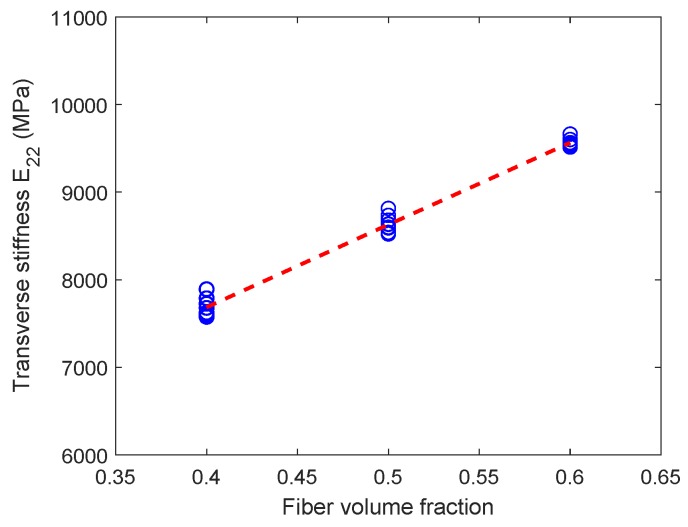
Effect of fiber volume fraction on the transverse stiffness for six-fiber unit cells. (D’Mello and Waas [12]).

**Figure 16:**
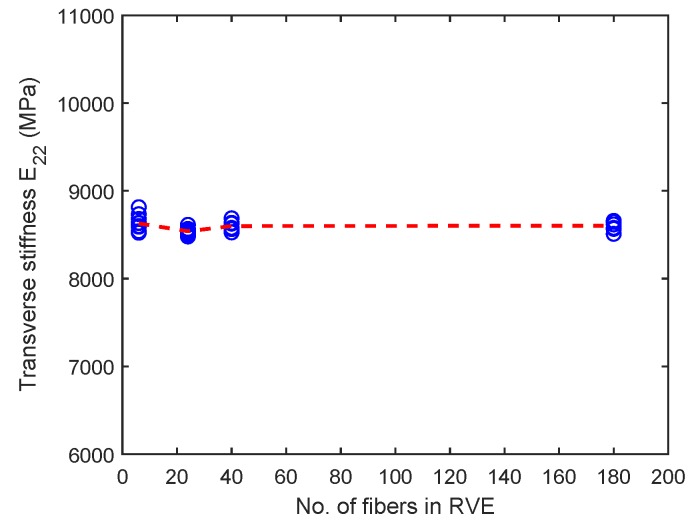
Variation of transverse stiffness with unit cell size with $V_{f}$ = 0.5. (D’Mello and Waas [12]).

Figure 15 shows the variation of transverse stiffness $E_{22}$ for the six-fiber unit cell case but for varying fiber volume fractions. As expected, there is a clear increase in the transverse stiffness for increasing fiber volume fraction. However, from Figure 16, no variation can be seen in the transverse stiffness of the unit cell as the unit cell size increases. This behavior is controlled by only the elastic properties of the constituents and is different from the observations in Figure 14 for the transverse tensile strength values, which depend on the matrix softening response. Moreover, the scatter in transverse tensile strength for a given type of unit cell is appreciably higher than scatter in transverse stiffness. This behavior can be explained due to the fact the stiffness is an average property of the constituents and is less sensitive to the distribution of fibers inside the unit cell, whereas the strength is controlled by extreme values of maximum principal stress developed in the matrix regions, which in turn, is controlled by fiber packing.

**Figure 17:**
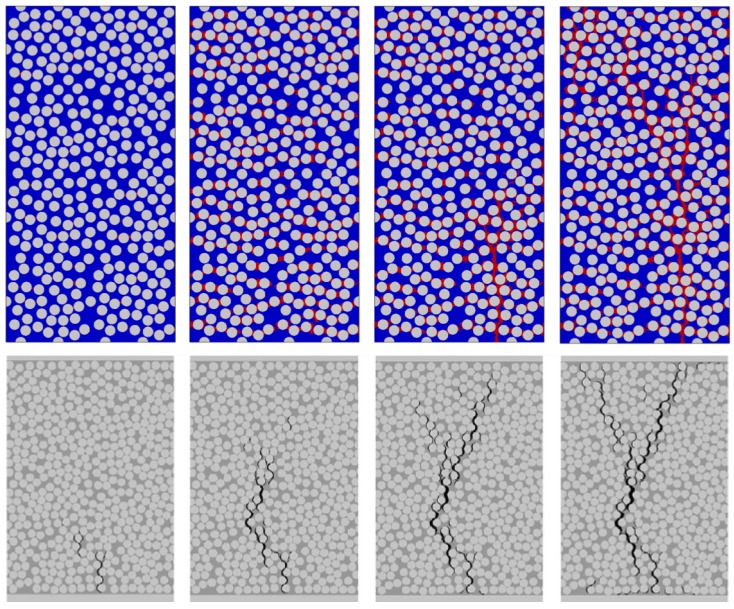
Qualitative comparison of two-piece matrix cracking from the present study (**top row**) with the computational study (**bottom row**) by Herráez et al. [19] on transverse cracking of an internal 90${}^{\circ }$ layer inside a [$0_{2}/90_{8}/0_{2}$] laminate.

Figure 17 shows a long 320-fiber unit cell subjected to transverse tension, starting with damage initiation to two-piece crack formation. Qualitative comparison is made with numerical results corresponding to a 90${}^{\circ }$ layer inside a [$0_{2}/90_{8}/0_{2}$] laminate (Herráez et al. [19]). In [19], in addition to matrix model, there was provision for fiber–matrix interface decohesion. It was reported that failure always initiated at the fiber–matrix interface and then this crack propagated and branched out through the thickness. In our simulations, the two-piece crack is seen to propagate similar to what is reported in [19]. In the present case, damage always occurred in the vicinity of the fiber, where there is stiffness mismatch between fiber and matrix properties and non-uniformity in maximum tensile stress field. Subsequent crack propagation through the thickness also showed the presence of crack-branching, which is also seen in the study by Herráez et al. [19].

### 3.2. Effect of Non-Uniformity in Matrix Tensile Strength on Unit Cell Transverse Tensile Strength

Apart from randomness in fiber distribution, non-uniformity in the matrix tensile strength can also influence the unit cell transverse strength. Another set of simulations were performed with normally distributed random variation of matrix strength within the unit cell, whereby the fracture toughness was kept constant. For mean strength μ and standard deviation *s*, we can generate a Gaussian random distribution $\sigma_{cr}∼N\left(\mu ,s^{2}\right)$. The first step is to generate two standard normal distributions N(0,1) with zero mean and two standard deviations $s_{1}=s_{2}=1$ from two independent *uniformly distributed* random variables $u_{1}$ and $u_{2}$, in [0, 1] respectively, using the Box–Muller transform [20]

(3) $$s_{1}=\left(-2\ln u_{1}\right)\cos\left(2\pi u_{2}\right)\;\mathrm{and}\;s_{2}=\left(-2\ln u_{1}\right)\sin\left(2\pi u_{2}\right).$$

Then the Gaussian distribution for quantity *y* of interest is obtained by the relation

(4) $$\sigma_{cr}=\mu +s_{1}s\;\mathrm{or}\;\sigma_{cr}=\mu +s_{2}s.$$

**Figure 18:**
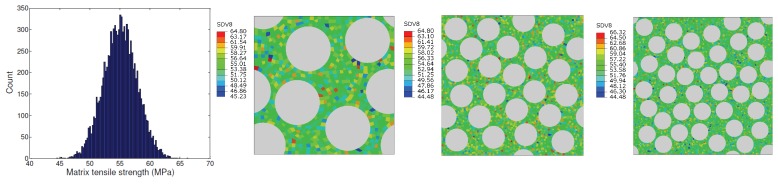
Unit cells with normally distributed tensile strength.

The ratio of the standard deviation to the mean $\left(\frac{s}{\mu }\right)$ is referred to as the coefficient of variation (COV). It is a non-dimensional measure of the dispersion of values about the mean. The distribution of mean matrix strength μ=55 MPa with COV = 0.05 assigned to integration points within the finite element model is shown in Figure 18. That is, the mean strength is identical to unit cells with uniform strength prescribed, which were discussed earlier.

**Figure 19:**
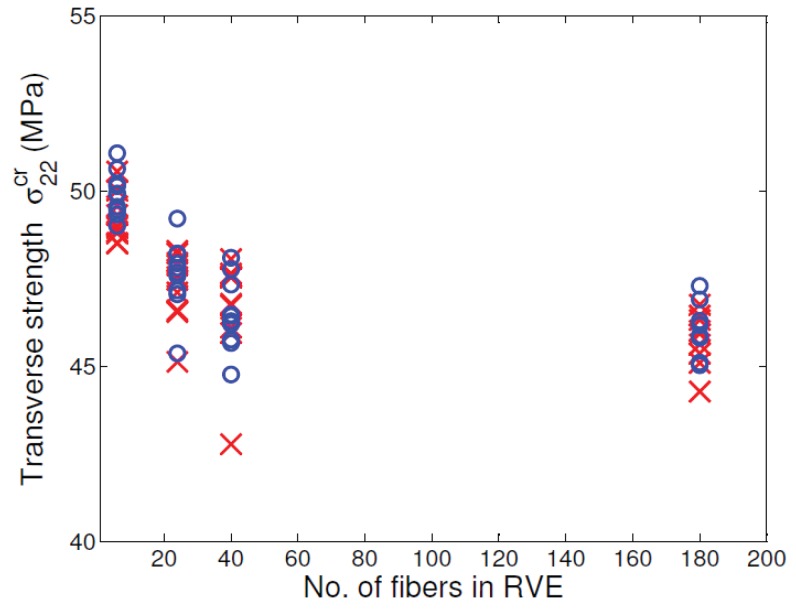
Comparison of transverse unit cell strength with variability in matrix tensile strength (red) with the case of uniform matrix tensile strength (blue), both cases as functions of unit cell size.

Figure 19 shows the variation of unit cell transverse tensile strength in the presence of normally distributed matrix strength (with mean strength μ= 55 MPa with COV = 0.05) along with the case when matrix strength is uniform (55 MPa). Introduction of non-uniformity of matrix tensile strength is seen to noticeably lower the unit cell transverse tensile strength. Even though some elements have strength greater than 55 MPa (up to 65 MPa), the values that are lower than the sample mean (those as low as 45 MPa) control the overall transverse tensile strength of the unit cell. Therefore, the unit cell strengths are seen to be sensitive to the lower tail of the prescribed strength distribution. This observation is consistent with the understanding that unlike stiffness, strength is controlled by extreme properties of the material. Note that since non-uniformity in strength was prescribed and elastic properties were kept uniform, the unit cell initial stiffness was not affected.

### 3.3. Effect of Unit Cell Size on Dissipation in the Matrix

**Figure 20:**
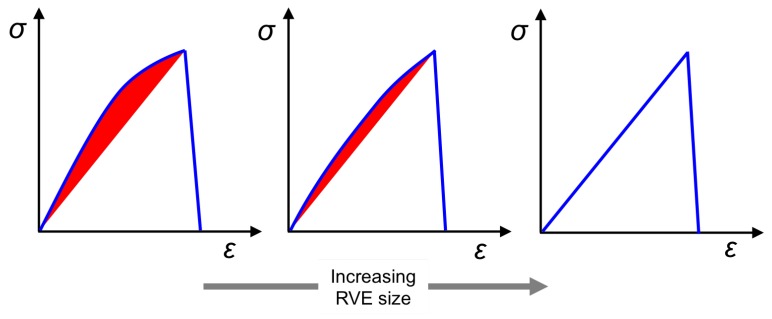
Schematic of the stress–strain response of unit cells with increase in unit cell size. The response of smaller unit cell sizes exhibit pronounced non-linearity owing dissipation in the matrix (shown in red shaded portion) before the peak.

The transverse tensile response of unit cells show a noticeable pre-peak nonlinearity for smaller sized unit cells, and this nonlinearity is observed to decrease with increase in the unit cell size. This pre-peak nonlinearity is seen as a reduction in the unit cell’s tangent stiffness $E_{22}$. This effect is schematically shown in Figure 20. With reference to Figure 20, the entire area under the global ε−σ diagram corresponds to the total energy dissipated during the fracture process. Note that, prior to the peak, since crack band has already been active for elements that are accumulating damage, the secant stiffness locally is assumed to govern the unloading behavior of such elements, as can be seen in unloading path of the constitutive law for damaging solid (Figure 6). Thus, it is assumed that upon unloading the unit cell just before the peak is encountered, the unit cell unloads all the way to the origin of the ε−σ diagram. However, the shaded area in red indicates the energy dissipated due to accumulation of damage just before failure (i.e., just before the peak in the ε−σ response) in the unit cell.

**Figure 21:**
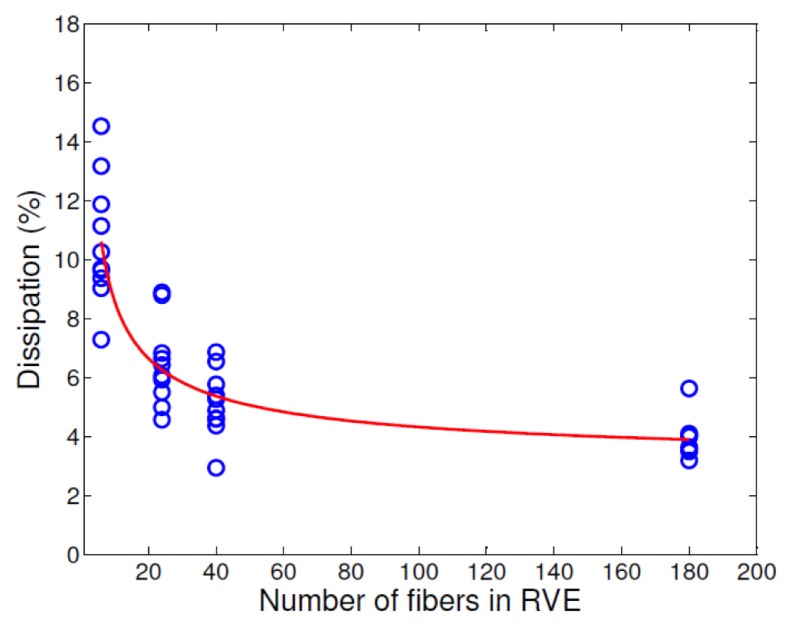
Percentage of energy dissipated (prior to peak) to the total energy dissipated in the unit cell, as a function of unit cell size.

In order to effectively quantify the dominance of the pre-peak nonlinearity to the overall dissipation, we can take the ratio of the pre-peak dissipation to the total dissipation in the unit cell. Note that for the case of no-dissipation prior to the peak, this fraction turns out to be zero since the unit cell transverse stiffness $E_{22}$ is constant up to the peak. For increasing unit cell sizes, the dissipation fraction, expressed as total percentage of the area under the ε−σ diagram is shown in Figure 21. The average value of this dissipation fraction drops rapidly for unit cells with six fibers to those having 40 fibers and this value stabilizes for larger sized unit cells. Thus, for a unit cell of fixed fiber volume fraction, with material properties of the constituents kept the same, the larger size unit cells exhibit brittle behavior compared to unit cells of smaller size. This observation also indicates that in larger size unit cells, dissipation occurs relatively in a localized manner compared to unit cells of smaller size.

The definition of an RVE, as presented in [18], is as follows; (i) an increase in its size does not lead to considerable differences in the homogenized properties, (ii) the microscopic sample is large enough so that the homogenized properties are independent of the microstructural randomness. In the results presented in this paper, it is seen that the converged unit cell size possesses these attributes, and therefore the converged unit cell size can be thought of as an RVE.

## 4. Summary and Future Work

Results have been presented for unidirectional fiber–matrix unit cells with randomly distributed fibers when loaded under transverse tension, in order to understand the effect of fiber packing and unit cell size on the mechanical properties such as transverse stiffness and transverse tensile strength. For a given unit cell type, the randomness in fiber packing was particularly seen to influence the scatter in transverse tensile strength values. Moreover, it was seen that for a fixed fiber volume fraction, the strength of the unit cell was inversely proportional to its size. However, with increase in unit cell size with fiber volume held fixed, there was no noticeable variation in the transverse stiffness values. The results from this investigation suggests that, when using homogenized properties from unit cells to be used at the global scale, care must be taken to ensure that sufficiently large unit cell must be chosen to ensure convergence of mean unit cell strength—else there is a possibility of over-predicting the homogenized strength. The practical application is primarily for use of quantifying stiffness and strength for a given fiber and matrix combination and also in determining converged unit cell size for such constituent combination, for use in homogenized finite element calculations. Moreover, analysis that includes randomness can provide bounds, especially for unit cell strength for use in design calculations. The influence of curing stresses for analysis conducted in this investigation on randomly packed unit cells is subject of ongoing research.
